# The Friend Within: Endophytic Bacteria as a Tool for Sustainability in Strawberry Crops

**DOI:** 10.3390/microorganisms10122341

**Published:** 2022-11-26

**Authors:** Ginaini Grazielli Doin de Moura, Aline Vieira de Barros, Franklin Machado, Caroline Marcela da Silva Dambroz, Chirlei Glienke, Desirrê Alexia Lourenço Petters-Vandresen, Eduardo Alves, Rosane Freitas Schwan, Moacir Pasqual, Joyce Dória

**Affiliations:** 1 Biology Department, Federal University of Lavras, Lavras 37200-900, Brazil; 2 Phytopathology Department, Federal University of Lavras, Lavras 37200-900, Brazil; 3 Phytopathology Department, Federal University of Viçosa, Viçosa 36570-900, Brazil; 4 Plant Genetics Department, Federal University of Lavras, Lavras 37200-900, Brazil; 5 Genetic Department, Federal University of Paraná, Curitiba 81531-980, Brazil; 6 Agriculture Department, Federal University of Lavras, Lavras 37200-900, Brazil

**Keywords:** plant-bacteria interaction, rhizobacteria, endophytic bacteria, biological nitrogen fixation, auxin, phosphate solubilization

## Abstract

Strawberry (*Fragaria* x *ananassa*, Duch.) is an important crop worldwide. However, since it is a highly demanding crop in terms of the chemical conditions of the substrate, a large part of strawberry production implies the application of large amounts of fertilizers in the production fields. This practice can cause environmental problems, in addition to increases in the fruit’s production costs. In this context, applying plant growth-promoting bacteria in production fields can be an essential strategy, especially thanks to their ability to stimulate plant growth via different mechanisms. Therefore, this study aimed to test in vitro and in vivo the potential of bacteria isolated from strawberry leaves and roots to directly promote plant growth. The isolates were tested in vitro for their ability to produce auxins, solubilize phosphate and fix nitrogen. Isolates selected in vitro were tested on strawberry plants to promote plant growth and increase the accumulation of nitrogen and phosphorus in the leaves. The tested isolates showed an effect on plant growth according to biometric parameters. Among the tested isolates, more expressive results for the studied variables were observed with the inoculation of the isolate MET12M2, belonging to the species *Brevibacillus fluminis*. In general, bacterial inoculation induced strain-dependent effects on strawberry growth. In vitro and in vivo assays showed the potential use of the *B. fluminis* MET12M2 isolate as a growth promoter for strawberries.

## 1. Introduction

Strawberry (*Fragaria ananassa*, Duch.) is a hybrid between the native species *Fragaria chiloensis* and *Fragaria virginiana* from the Rosaceae family [1], and its (pseudo) fruit is appreciated worldwide for its organoleptic and nutraceutical characteristics [2,3]. FAO data indicate that the world production of strawberries exceeds 9 million tons in a cultivated area of close to 400 thousand hectares from tropical, subtropical, and temperate zones [4]. The best productivity regarding strawberry production has been achieved through intensive systems, especially with the application of highly soluble chemical fertilizers in productive fields, since this crop is highly demanding in terms of soil chemical fertility [5].

The impacts of these practices on the environment and on food production costs have been described [6]. By the year 2050, the expected growth of the world’s population will require an estimated increase of around 60% in agricultural production compared to current production levels. Consequently, this will result in the intensification of production systems [7,8,9]. Moreover, changes in production strategies are necessary in this scenario, especially in the search for environmentally correct and economically viable ways of producing food. In ecological food systems, concepts that emphasize biological interactions among its components are employed. These systems are influenced by and count on interactions developed in the rhizosphere at the interface between root and soil that is under the direct influence of root exudates. This is also a zone of intense microbial activity, concentration, and diversity [10,11,12,13,14,15,16,17].

Plant growth-promoting bacteria (PGPB) can be defined as microorganisms present in the rhizosphere that are able to develop harmonious interactions with plants, modulating their metabolism and stimulating their productivity [18,19]. PGPR can hold great potential for application in agriculture thanks to their direct plant-growth promotion abilities, being used as biofertilizers [20,21,22,23,24,25], phytostimulators [26,27,28,29] and rhizomediators (induction of the plant tolerance to contaminants) [30]. In addition, they stimulate tolerance to environmental stress, such as low soil fertility [31], heavy metal contents [32] or drought [33,34,35].

The use of PGPB has been studied in strawberry crops. The colonization of strawberry roots by *Azospirillum brasilense* has already been reported [36,37,38]. After inoculating some PGPBs on strawberry plants, Pereira et al. [39] demonstrated the effect of a wide variety of microorganisms with different physiological and biochemical capacities. In addition, some studies have shown efficient growth promotion of strawberry plants after inoculation with PGPB [38,39,40,41,42,43,44]. New research work testing the inoculation of PGPBs in strawberries can add important information about the potential use and benefit of new efficient bacterial strains.

Based on the presented context, this work aimed at evaluating the potential for direct plant-growth promotion of bacteria isolated from strawberry leaves and roots in vitro and in vivo.

## 2. Materials and Methods

### 2.1. Bacterial Isolation and Culture

The bacteria used in this work were previously isolated from strawberry leaves and roots of cv. Aromas, as described by Andrade [45].

Bacterial isolates were reactivated in a nutrient-agar medium (3 g·L^−1^ meat extract, 5 g·L^−1^ peptone, 15 g·L^−1^ agar). For the auxin production tests and the inoculant production, the isolates were cultivated in a nutrient broth medium (3 g·L^−1^ of meat extract, 5 g·L^−1^ of peptone).

In order to evaluate the biological nitrogen fixation capacity, the isolates were cultivated in nitrogen-free bromothymol blue (NFb) medium (5 g·L^−1^ of malic acid, 0.5 g·L^−1^ of K_2_HPO_4_, 0.2 g·L^−1^ of MgSO_4_7H_2_O, 0.1 g·L^−1^ of NaCl, 0.02 g·L^−1^ CaCl_2_2H_2_O, 1 mL vitamin solution, 2 mL micronutrient solution, 4 mL 1.64% FeEDTA, 2 mL bromothymol blue solution, 4.5 g·L^−1^ KOH and 1.8 g·L^−1^ agar) modified by the addition of 5 g·L^−1^ sucrose. For the production of this medium, the vitamin solution consisted of 10 mg of biotin and 20 mg of pyridoxol-HCl in 100 mL of sterile distilled water (SDW); the micronutrient solution was composed of 0.2 g of Ca_2_MoO_4_2H_2_O, 0.235 g of MnSO_4_H_2_O, 0.28 g of H_3_BO_3_, 0.008 g of CuSO_4_5H_2_O and 0.024 g of ZnSO_4_. 7H_2_O in 200 mL of SDW, and the blue solution of bromothymol was obtained at 0.5% in 0.2N KOH [46].

To evaluate the capacity to solubilize phosphate and calcium, the isolates were cultivated in NBRIP medium (10 g·L^−1^ of glucose, 5 g·L^−1^ of Ca_3_(PO_4_)_2_, 5 g·L^−1^ of MgCl_2_6H_2_O, 0.25 g·L^−1^ of MgSO_4_ 7H_2_O, 0.2 g·L^−1^ KCl, 0.1 g·L^−1^ (NH_4_)2SO_4_ [47]), to which 15 g·L^−1^ agar was added.

In all tests, the isolates were incubated at 30 °C for growth and, when cultivated in liquid medium, were kept under constant agitation of 120 rpm in a shaker under the same conditions mentioned above.

### 2.2. In Vitro Evaluation of Plant Growth-Promoting Potential

#### 2.2.1. Biological Nitrogen Fixation Capacity Test

The asymbiotic biological nitrogen fixation capacity of the bacterial isolates was tested using the method proposed by Dobereiner [46]. For this purpose, the isolates were cultivated in NFb medium modified by adding 5 g·L^−1^ of sucrose, with pH adjusted to 6.8–7.0.

Bacterial isolates were previously activated in a nutrient-agar culture medium for 48 h. Then, they were cultured in the modified semi-solid NFb medium by stab inoculation.

The strains were incubated at 30 °C and evaluated every day after inoculation for 10 days. Isolates were considered capable of biologically fixing nitrogen when a halo or a typical aerotaxic film was observed near the surface of the culture medium, indicating a reduction of atmospheric nitrogen into ammonia. The strain Ab-V5 of *Azospirillum brasiliense* was used as the positive control. Analyses were performed in a triplicate for each isolate, with one tube considered a biological replicate.

#### 2.2.2. Auxin Production Capacity Test

The ability of the bacterial isolates to biosynthesize indole-acetic acid was confirmed by the Salkowski colorimetric method [48,49]. Therefore, the isolates were cultivated in nutrient broth medium and incubated at 30 °C for 48 h. After this period, the concentration of bacterial cells was adjusted to 10^8^ cells/mL (OD_600_ = 0.5, based on the McFarland scale).

Subsequently, 10% (*v*/*v*) of the bacterial culture was transferred to freshly prepared nutrient broth medium supplemented with tryptophan (100 µg/mL). The tubes were incubated at 30 °C for 72 h in the dark under constant agitation at 120 rpm. After this period, auxin production was determined by mixing the recovered supernatant with Salkowski’s Reagent (1.875 g of FeCl_3_6H_2_O, 150 mL of 35% H_2_SO_4,_ and 100 mL SDW). After incubation of the mixture at 30 °C for 15 min, absorbance was measured at a wavelength of 530 nm. The quantification of auxin production was performed by comparison to a standard curve obtained from an IAA (indole acetic acid) SDW solution. The reddish-pink color of the samples was considered indicative of auxin production. For this evaluation, the strain Ab-V5 *Azospirillum brasiliense* was used as a positive control. All analyses were performed in triplicate for each isolate, with one tube considered a biological replicate.

#### 2.2.3. Calcium and Phosphate Solubilization Test

The evaluation of the phosphate solubilization capacity of the isolates was carried out using the methodology proposed by Nautiyal [47].

Initially, the isolates were cultivated in a nutrient broth medium with pH 6.8–7.0 for 48 h. After this period, the density of bacterial cells in the solution was adjusted to 10^8^ cells/mL according to the McFarland scale. A 10µL aliquot of the bacterial suspension was inoculated into Petri dishes containing NBRIP culture medium. All strains were incubated at 30 °C, and the evaluation was carried out every 3 days until the 12th day after inoculation.

The isolates were considered capable of solubilizing inorganic and insoluble phosphate when a translucent halo around the colonies was observed (translucent surrounding area, solubilization area). The diameter of the translucent halo was measured, and on the 12th day, the phosphate solubilization index was calculated using the ratio between the diameter of the halo and the diameter of the colony [50,51]. *Azospirillum brasilense* Ab-V5 was used as a positive control for this test. The evaluations were carried out in triplicate, with each Petri dish considered a biological replicate.

### 2.3. Evaluation of Direct Growth Promotion Ability in Strawberry Plants

Auxin production and phosphate solubilization data were evaluated using multivariate statistical analysis (Supplementary data, Table S1). The bacterial strains showing the highest scores were selected for further tests as long as they also showed an in vitro asymbiotic nitrogen fixation capacity. In addition, isolates that belonged to genera of human pathogens or environmental contaminants were discarded. Five bacterial strains were selected and evaluated for their capacity to promote plant growth in strawberries in two different assays conducted in a greenhouse under a completely randomized design (with 10 biological replicates, one seedling per treatment).

To this end, each isolate was considered a different treatment. A negative control with 30% nitrogen and phosphorus but without bacterial inoculation was also added. Moreover, a positive control with the application of a complete nutrient solution and a control with inoculation of *Azospirillum brasilense* Ab-V5 were added as well. In both assays, strawberry seedlings of the cv. Aromas obtained in tissue culture were cultivated in a previously sterilized inert substrate composed of sand and vermiculite in a 1:1 (*v*:*v*) ratio. In addition, Algerian natural phosphate with 29% P_2_O_5_ and solubility equal to 2% in citric acid was added to the substrate in all treatments. Therefore, phosphate was calculated to supply 260 kg/ha of P_2_O_5_ [5].

The seedlings were irrigated every 2 days with SDW. In addition, every 5 days, Hoagland and Arnon nutrient solution was applied (complete for the positive control, and 30% nitrogen and phosphorus for treatments with inoculation of bacterial isolates and negative control) was applied. The complete solution of Hoagland and Arnon [52] used for the treatment without inoculation + complete fertilization was composed of 1 mL^−1^ of KH_2_PO_4_ (1 mol/L), 5 mL^−1^ KNO_3_ (1 mol/L), 5 mL^−1^ of Ca(NO_3_)_2_ (1 mol/L), 2 mL^−1^ of MgSO_4_ (1 mol/L), 1 mL of micronutrient solution (2.86 g·L^−1^ of H_3_BO_3_, 1.81 g·L^−1^ MnCl_2_, 0.22 g·L^−1^ of ZnSO_4_7H_2_O, 0.08 g·L^−1^ of NaMoO_4_H_2_O) and 1 mL of Fe-EDTA (24.1 g·L^−1^ of FeSO_4_7H_2_O and 25.1 g·L^−1^ of EDTA). The solution with reduced addition of nitrogen and phosphorus (30% of total) was prepared by replacing KH_2_PO_4_, KNO_3_ and Ca(NO_3_)_2_ with 5.7 mL L^−1^ of KCl (1 mol/L), 5 mL L^−1^ of CaCl_2_ (1 mol/L), 0.3 mL L^−1^ of KH_2_PO_4_ (1 mol/L) and 3 mL L^−1^ of NH_4_NO_3_ (1.5 mol/L) [53].

The bacterial inoculum was previously obtained by cultivating selected strains in nutrient broth medium at 30 °C under constant agitation for 48 h and subsequent adjustment of cell density to 1 × 10^8^ cells/mL.

The growth of strawberry seedlings was evaluated five months after the inoculation with the selected bacterial strains. Root length, root and shoot dry mass (after conditioning the material in a forced circulation oven at 60 °C until the weight stabilizes), total dry mass, number of leaflets, leaf area, and content of nitrogen and phosphorus in dry mass were measured [54].

### 2.4. Number of Leaves and Leaf Surface Measurements

The total number of leaves of each individual strawberry plant was measured at the end of five months of culture. After collecting leaves, three of them were scanned per individual plant. The measure of the leaf area was obtained considering its contour with the help of ImageJ software (https://imagej.nih.gov/ij/, accessed on 10 June 2019).

### 2.5. Quantification of Nitrogen Content in Leaves

The leaf nitrogen content of plants subjected to treatments with reduced fertilization + no inoculation, complete fertilization, and inoculation of selected isolates was quantified at the end of the fourth month of the assay.

The leaf nitrogen content was determined in all samples using the H_2_SO_4_ + H_2_O_2_ digestion method and then distilled using the Kjeldahl method, as described by Silva [54]. The nitrogen content accumulated in the plants was then calculated by multiplying the concentration of the element by the respective dry matter weight.

### 2.6. Quantification of Leaf Phosphorus Content

After inoculating strawberry plants with the plant-growth-promoting bacteria selected in vitro, the leaf phosphorus content was quantified.

To this end, plants were submitted to H_2_SO_4_ + H_2_O_2_ digestion. Then, the determination of the total leaf phosphorus content was performed by the molybdenum blue spectrophotometry method, as described by Silva [54].

The total leaf phosphorus content was obtained by comparing the absorbance at 420 nm of the samples on a standard curve produced from the absorbance measurement of standard solutions with known phosphorus concentrations.

The phosphorus content accumulated in the leaves was then calculated by multiplying the concentration of the element by the respective shoot dry matter weight.

### 2.7. Phylogenetic Analysis Based on the 16S rDNA Gene

Bacterial isolates that showed the most significant plant growth-promoting potential were identified by sequencing the 16S rDNA ribosomal gene. The genomic DNA was extracted from 10 mL of bacteria cultured for 48 h under agitation on a liquid medium. The Wizard Genomic DNA Purification Kit (Promega, MD, Madison, WI, USA) was used for extraction, following the protocol indicated by the manufacturer with minor modifications. The quantity and purity of the DNA were measured in a spectrophotometer (NanoVuePlus GE Healthcare, Munich, Germany) by observing the nucleic acid concentrations and the proportions of purity at A260/280 and A260/230, prioritizing values between 1.80 and 2.20. DNA integrity was also visually evaluated on 1% agarose gels and TAE buffer (Tris, EDTA, and boric acid).

For PCR amplification of the 16S rDNA, three primers were used in two combinations: two forwards, 27 (AGA GTT TGA TCM TGG CTC AG) and 515 (GTG CCA GCM GCC GCG GTA A), and one reverse, 1492 (CGG TTA CCT TGT TAC GAC TT) [55]. The PCR reactions were performed with a final volume of 30 µL, adding 6.0 µL of the 5 FIREPol Master Mix (12.5 mM MgCl_2_, 0.4 M Tris-HCL, 0.1 M (NH_4_) 2SO_4_, 0.1% *w*/*v* Tween-20 and 1 mM dNTPs of each nucleotide), 0.9 µL of the forward primer (10 mM) and 0.9 µL of the reverse primer (10 mM), 19.7 µL of free nuclease H_2_O and 2.5 µL of DNA (10 ng/µL). The reactions were carried out in a SimpliAmp thermocycler with the following settings: 2 min at 95 °C for initial denaturation, 30 cycles at 95 °C for 30 s, 55 °C for 30 s and 72 °C for 100 s, ending with 72 °C for 5 min and 4 °C until electrophoresis on 1% agarose gel.

The amplified gene segments were purified with sodium acetate 3 M and ethanol 95% using 0.1 and 2.5× the reaction volume used, respectively. The purified products were confirmed by electrophoresis on 1% agarose gel and used for the Sanger reaction, performed with the BigDye terminator (PE Applied Biosystems, Waltham, MA, USA), and primers 27, 515, and 1492, separately, according to the manufacturer’s protocol. The ABI 3730xL DNA ANALYZER 48 capillary sequencer (PE Applied Biosystems, Waltham, MA, USA) was used to read the samples.

The consensus sequences were processed in Bioedit 7.2.5 software (http://en.bio-soft.net/format/BioEdit.html, accessed on 17 December 2021) and compared to sequences available in the NCBI/GenBank database (National Center for Biotechnology Information-http://www.ncbi.nlm.nih.gov/BLAST/, accessed on 17 December 2021) using the Blast tool, limiting to the sequences of the type strains. The genera found were examined in the LPSN, or the List of Prokaryotic names with Standing in Nomenclature (https://lpsn.dsmz.de/, accessed on 17 December 2021), and the validated species were used in the alignment. The alignment was performed using MAFFT ver. 7 [56] with the auto option and best-fit substitution models were selected for each alignment using ModelFinder [57] according to the corrected Akaike criterion. Phylogenetic trees were built with maximum likelihood (ML) and Bayesian inference (BI) approaches. For ML analyses in IQ-TREE2 [58,59], branch support values were obtained with the ultrafast bootstrap method and the SH-aLRT branch test using 1000 replicates. BI analyses were performed in MrBayes ver. 3.2.7 [60], using two parallel runs with one cold and three heated chains each, using the number of generations required to reach a standard deviation of split frequencies of ≤0.01 and a sampling frequency set to every 10,000 generations. The posterior probability values were calculated after discarding the first 25% of the generated trees as burn-in. The resulting trees were plotted in FigTree ver. 1.4.2 (http://tree.bio.ed.ac.uk/software/figtree/, accessed on 17 December 2021). All sequences obtained were deposited in the GenBank database with the access codes listed in Table 1. The final trees and alignments are deposited in TreeBASE (study number S29343).

### 2.8. Statistical Analysis

Results obtained were subjected to analysis of variance, comparing the means in R [61] using the Scott–Knott test (ExpDes package) at 5% probability.

To select bacteria from in vitro growth promotion tests, principal component analysis (PCA) was performed using the average of three replicates for each variable (phosphate solubilization and auxin production). Therefore, vectors and estimated eigenvalues were obtained for each principal component (CP) using a correlation matrix. The scores of each isolate were calculated from the eigenvectors of the first CP. The bacterial isolates with the highest scores were selected for further in vivo testing. The analysis was performed using a statistical computing environment R [61]. All graphics were produced with SigmaPlot 13.0 [62].

## 3. Results

### 3.1. Auxin Biosynthesis

**Figure 1:**
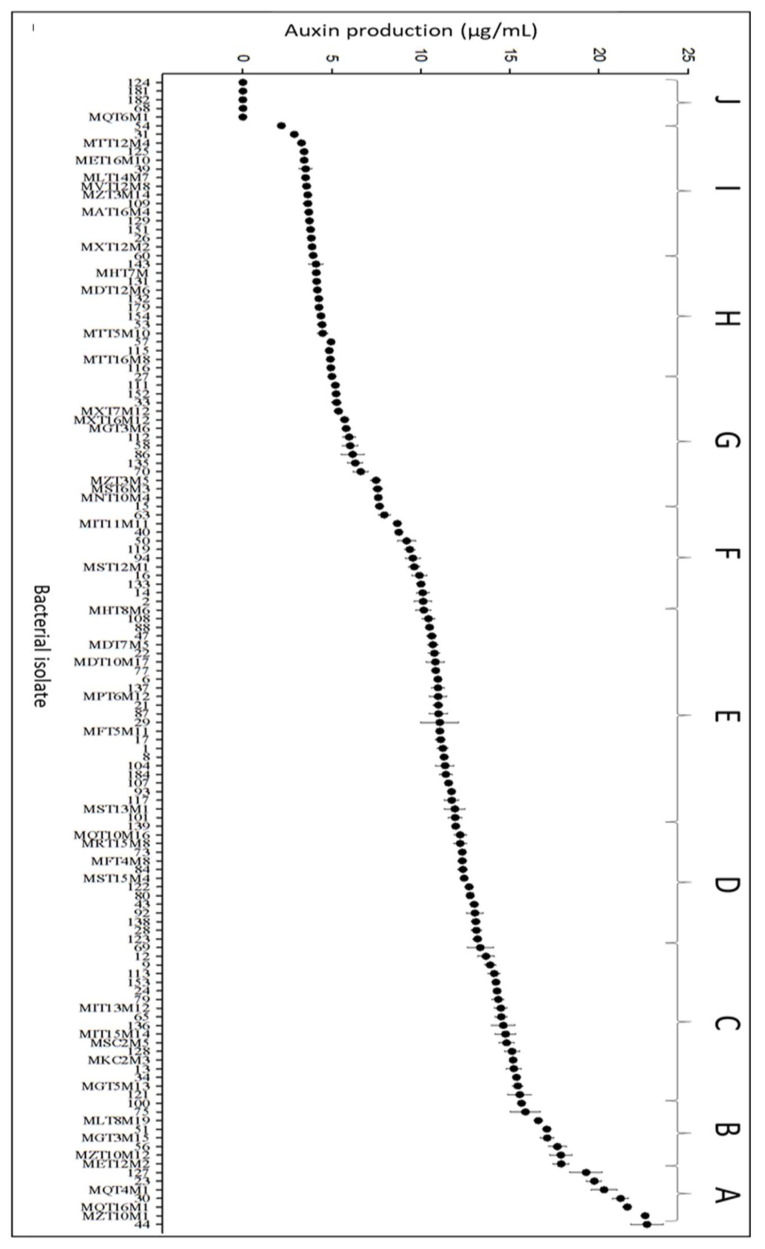
Biosynthesis of auxins by strawberry endophytic bacteria. Different letters differ significantly from each other by the Scott–Knot test at 5% probability.

Results obtained in the in vitro auxin biosynthesis tests showed the ability of bacterial isolates to produce this phytohormone (Figure 1). Among all tested bacterial strains, 130 showed some level of auxin production. There was a considerable variation in this metabolite production among the isolates, with values ranging from 0 (for isolates 124, 181, 182, 68, and MQT6M1) to 231 µg/mL (for isolate 44). The highest auxin production values were observed in treatments with isolates 44, MZT10M1, MQT16M1, 30, MQT4M1, 23, and MET12M2 (Figure 1).

### 3.2. Phosphate Solubilization

**Figure 2:**
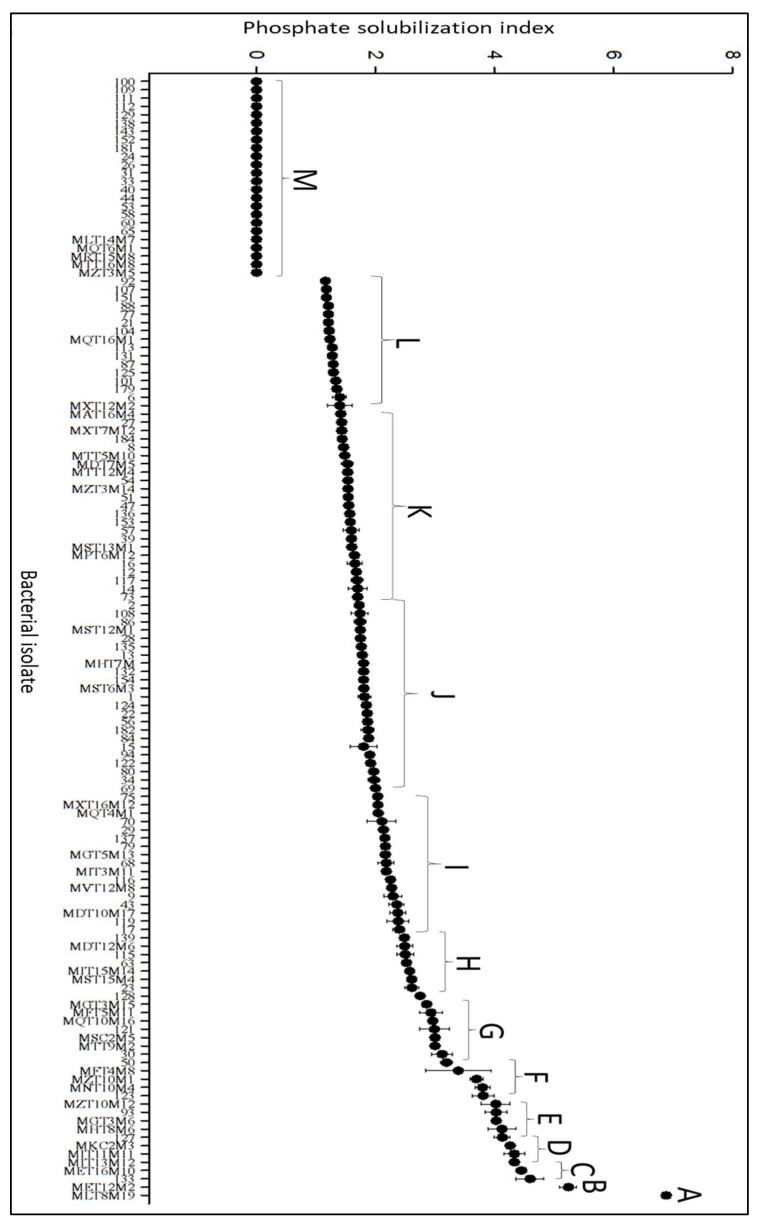
Phosphate solubilization by strawberry endophytic bacteria. Different letters differ significantly from each other by the Scott–Knot test, at 5% probability.

Results obtained in the in vitro phosphate solubilization assays suggested that several bacterial isolates have this ability (Figure 2). There was a significant variation in the solubilization of this element in the culture medium among the isolates, with the phosphate solubilization index values found to vary in the experiments from 0 to 7 (for the MLT8M19 isolate). The highest levels of phosphate solubilization were observed in treatments with isolates MLT8M19, MET12M2, MET16M10, MIT13M12, and MKC2M3 (Figure 2).

### 3.3. Biological Nitrogen Fixation BNF

Only 59 isolates presented the ability to asymbiotically fix nitrogen belonging to different genera such as *Enterobacter*, *Rhizobium*, *Pantoea*, *Acinetobacter*, *Bacillus*, *Paenibacillus*, *Pseudomonas,* and *Burkholderia*. However, since this test is qualitative, no inference about the quantity of fixed nitrogen was possible (Table 2).

### 3.4. Phylogenetic Analysis

**Figure 3:**
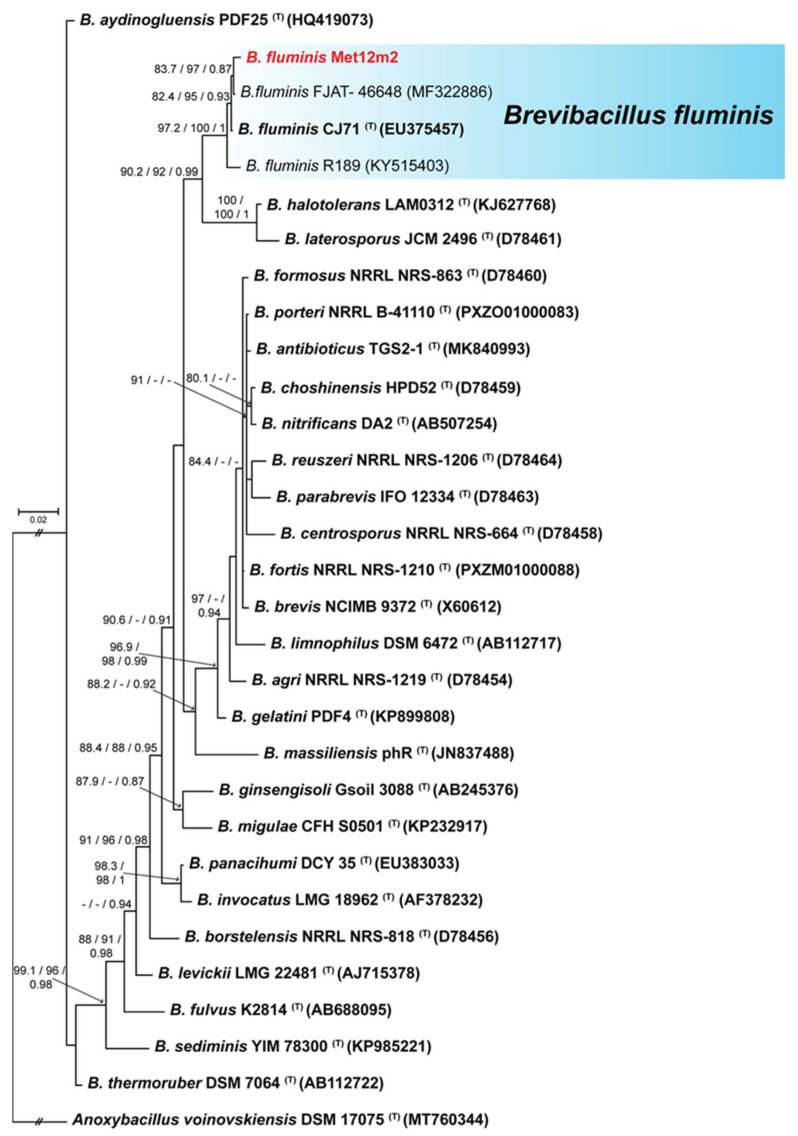
Bayesian inference phylogenetic tree of MET12M2 isolate and 27 species of *Brevibacillus* based on 16S rDNA sequences. The species *Anoxybacillus voinovskiensis* (DSM17075) was used as outgroup. Strains marked with a “T” and emphasized in bold correspond to sequences from type-strains. The scale bar of 0.02 represents the number of changes per site. MLsh-ALRT, ultrafast bootstrap values, and posterior probability support values are presented on the left side of nodes.

**Figure 4:**
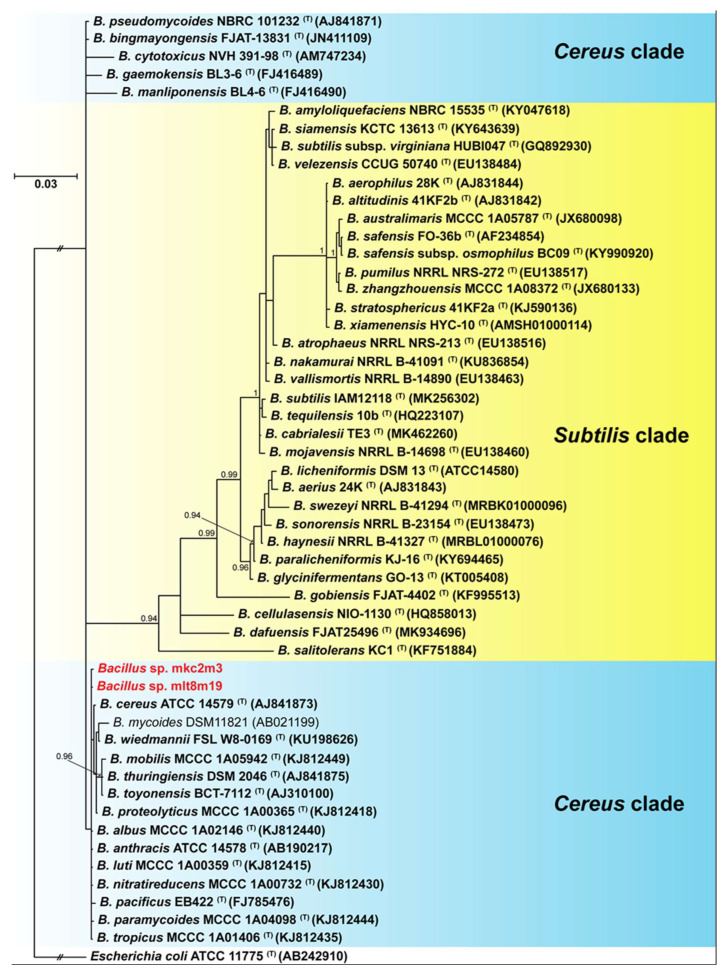
Bayesian inference phylogenetic tree of MKC2M3 and MLT8M19 isolates and all accepted species of *Bacillus* based on the 16S rDNA sequences. The species *Escherichia coli* (ATCC11775) was used as outgroup. Strains marked with a “T” and emphasized in bold correspond to sequences from type-strains. The scale bar of 0.03 represents the number of changes per site. MLsh-ALRT, ultrafast bootstrap values, and posterior probability support values are presented on the left side of nodes.

**Figure 5:**
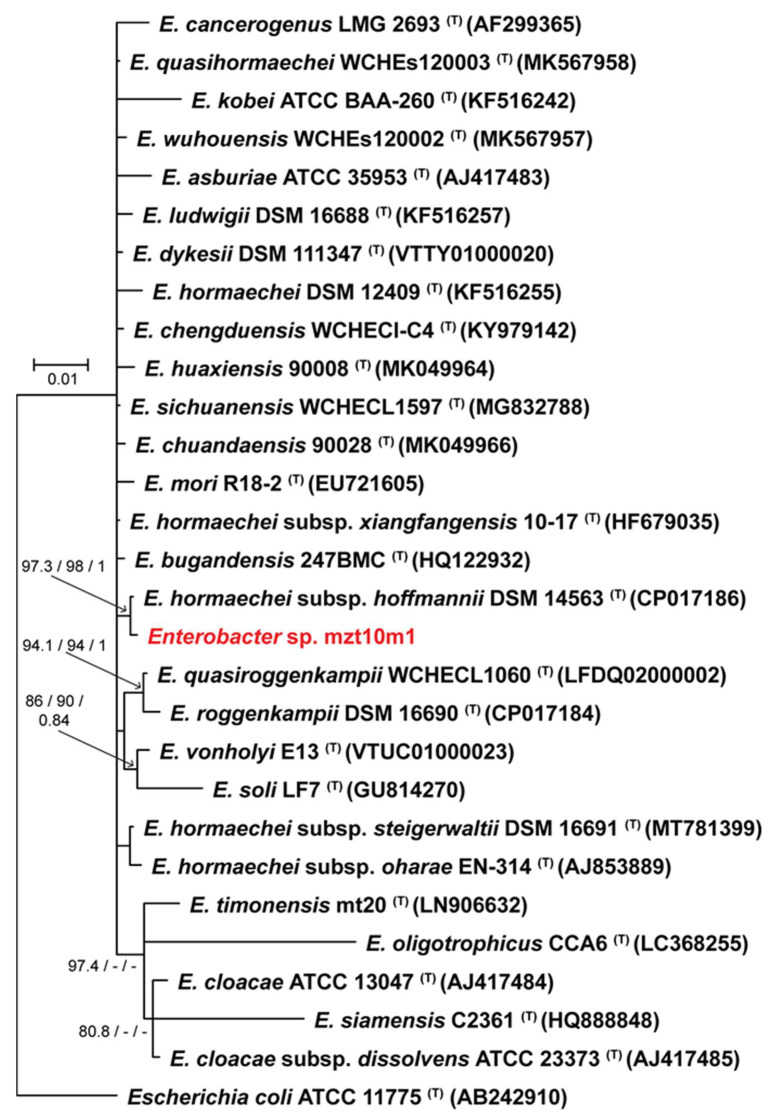
Bayesian inference phylogenetic tree of MZT10M1 isolate and all accepted species of *Enterobacter* based on the 16S rDNA sequences. The species *Escherichia coli* (ATCC11775) was used as outgroup. Strains marked with a “T” and emphasized in bold correspond to sequences from type-strains. The scale bar of 0.01 represents the number of changes per site. MLsh-ALRT, ultrafast bootstrap values, and posterior probability support values are presented on the left side of nodes.

**Figure 6:**
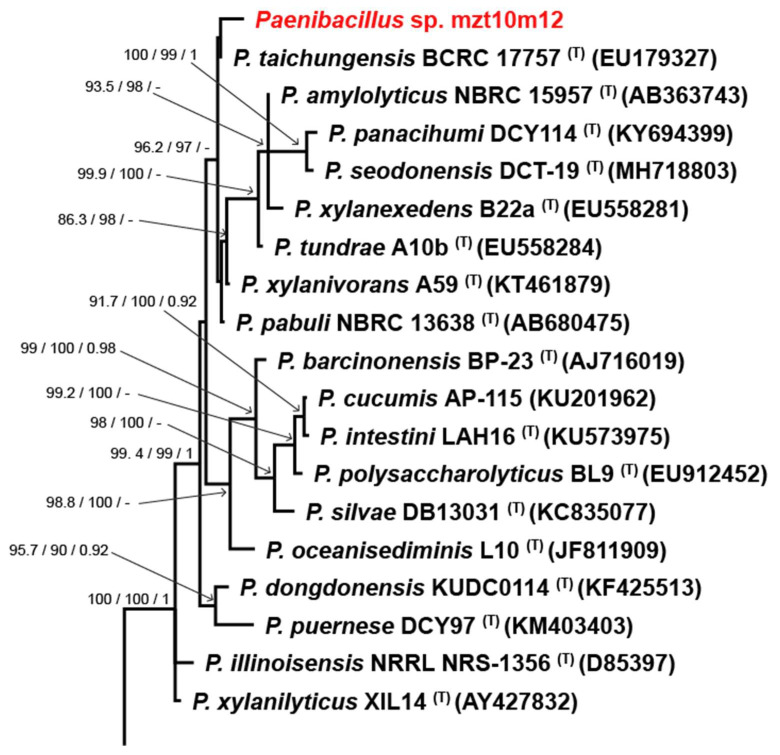
Segment of the Bayesian inference phylogenetic tree of MZT10M12 isolate and all accepted species of *Paenibacillus* based on the 16S rDNA sequences. The species *Paenibacillus selenitireducens* (ES3-24) was used as outgroup. Strains marked with a “T” and emphasized in bold correspond to sequences from type-strains. The scale bar of 0.01 represents the number of changes per site. MLsh-ALRT and ultrafast bootstrap values, and posterior probability support values are presented on the left side of nodes. The complete phylogenetic tree of MZT10M12 is available in the Supplementary Material.

MET12M2, MKC2M3, MLT8M19, MZT10M1, and MZT10M12 isolates showed the most significant plant growth-promoting potential and were selected for further tests. These isolates were also identified (Table 2) as belonging to *Brevibacillus fluminis* (MET12M2—Figure 3), *Bacillus* sp. Cereus clade (MKC2M3 and MLT8M19—Figure 4), *Enterobacter* sp. (MZT10M1—Figure 5), and *Paenibacillus* sp. (MZT10M12—Figure 6).

This analysis allowed the identification of 4 major groups where the five bacterial isolates were distributed (Figure 3, Figure 4, Figure 5 and Figure 6). The 16S rDNA sequence allowed the identification of the MET12M2 isolate at the species level, as this isolate clustered to the type strain of *B. fluminis* with high support values (Figure 3).

Isolates MKC2M3 and MLT8M19 were identified as *Bacillus* sp. Cereus clade (Figure 4). However, additional gene sequences are needed to identify these isolates at the species level. Isolate MZT10M1 grouped into the *Enterobacter hormaechei* subsp. *hoffmannii*-type strain with a high support value (Figure 5). However, as several other *Enterobacter hormaechei* subspecies were grouped outside this cluster and together with other *Enterobacter* species, we identified MZT10M1 as *Enterobacter* sp., as the addition of sequences from other genes is needed to confirm the species identification.

Isolate MZT10M12 was identified as *Paenibacillus* sp. (Figure 6), given the limitations of 16S rDNA-based phylogenetic analysis due to the presence of different copies of the 16S rDNA gene in several species of *Paenibacillus* [63,64]. According to the literature, up to 10 copies of the 16S rDNA gene can be found in the genome of different *Paenibacillus* species, causing ambiguous results due to sequence heterogeneities [63,65,66,67]. The use of *gyrB*, *recA*, *recN*, and *rpoB* genes and genome analysis was suggested as an alternative to the 16S rDNA gene for *Paenibacillus* species [68,69].

### 3.5. Growth Promotion of Strawberry Plants

**Figure 7:**
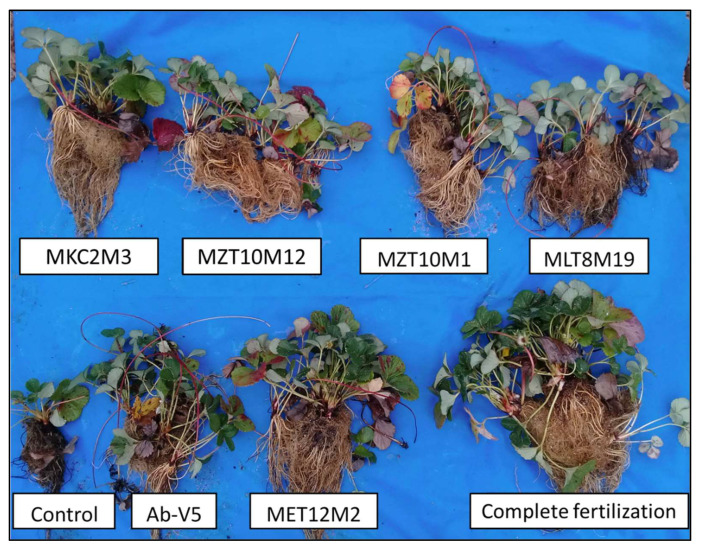
Growth of inoculated and non-inoculated strawberry plants. Data below each plant refer to the treatments applied. From left to right, and top: inoculation with isolate MKC2M3 (*Bacillus* sp. Cereus clade), inoculation with MZT10M12 (*Paenibacillus* sp.), inoculation with MZT10M1 (*Enterobacter* sp.), inoculation with MLT8M19 (*Bacillus* sp. Cereus clade), control with reduced N and P, inoculation with Ab-V5 (*Azospirillum brasilense*), inoculation with MET12M2 (*Brevibacillus fluminis*) and complete fertilization.

Isolates MLT8M19, MKC2M3 (*Bacillus* sp. Cereus clade), MET12M2 (*Brevibacillus fluminis*), MZT10M1 (*Enterobacter* sp.), and MZT10M12 (*Paenibacillus* sp.) were selected for further assays. The inoculation of strawberry seedlings with bacterial isolates previously selected in vitro caused changes in plant growth (Figure 7).

**Figure 8:**
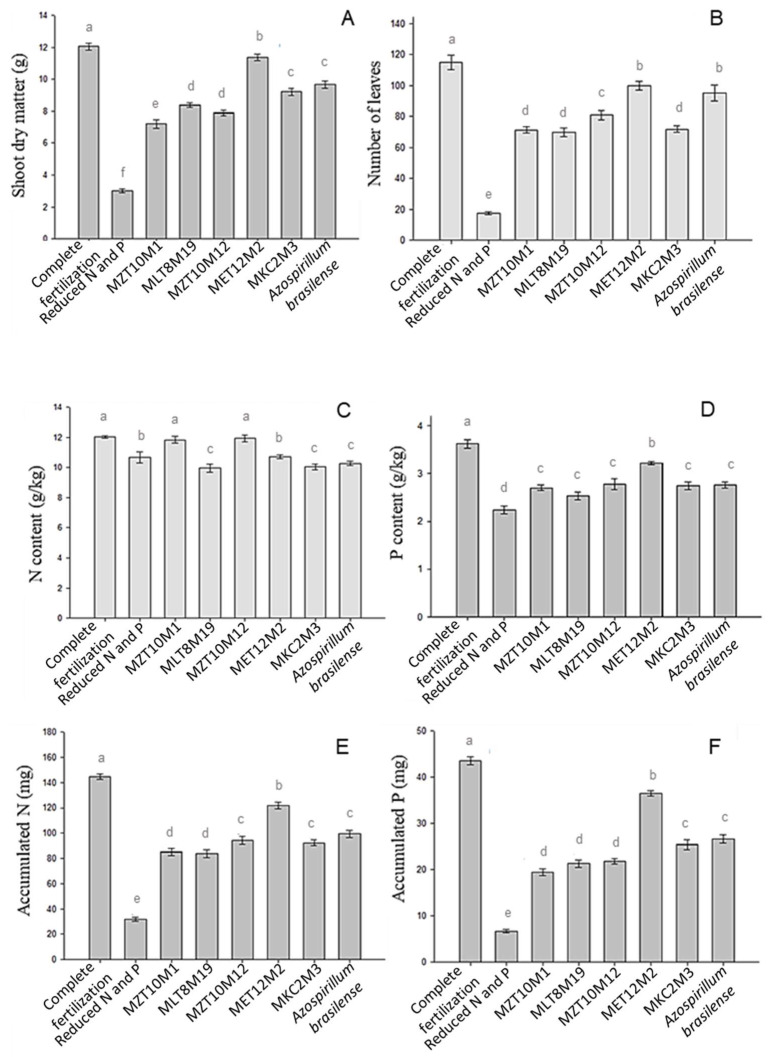
Plant dry matter (**A**), number of leaves (**B**), leaf N content (**C**), leaf P content (**D**), accumulated N (**E**), and accumulated P (**F**) of plants uninoculated or inoculated with in vitro selected endophytic bacteria. Means represented by the same letter do not differ significantly by the Scott–Knot test at 5% probability.

Shoot dry matter (Figure 8A) was significantly influenced by treatments (*p* < 0.05). The highest production of dry matter was observed with complete fertilization, with an increase of 301.67% in shoot dry matter production. Treatment with inoculation of isolate MET12M2 allowed an increase of 279% for the same variable. Other bacterial isolates provided slight or intermediate increases in shoot dry matter, but all treatments differed statistically from the control, with nitrogen and phosphate fertilization reduced by 70% for no inoculation.

A similar result was observed for the number of leaves (Figure 8B). The application of complete mineral fertilization induced an increase of 556.57% in this variable, significantly different from all other treatments (*p* < 0.05). The inoculation of Ab-V5 (*Azospirillum brasilense*) and the MET12M2 differed from other treatments, increasing 444% and 470.86% compared to the control, respectively. Other isolates showed intermediate or low effects on the production of leaves, but they differed significantly from the control with reduced fertilization and no inoculation.

The leaf nitrogen (Figure 8C) and phosphorus (Figure 8D) contents of strawberry plants were also significantly influenced by the treatments (*p* < 0.05). The highest values of leaf nitrogen content were obtained through the application of complete mineral fertilization and inoculation of MZT10M1 and MZT10M12, which did not significantly differ from the control. The complete mineral fertilization presented 12.02 g of N/kg of plant, whereas plants inoculated with MZT10M1 and MZT10M12 had contents of 11.84 g N/kg of plant and 11.94 g N/kg of plant, respectively. Therefore, the application of complete mineral fertilization and inoculation of isolates MZT10M1 and MZT10M12 increased by 12.55%, 10.86%, and 11.8% in this variable, respectively, compared to the reduced fertilization and no inoculation.

Inoculation with MET12M2 was not significantly different from the control with reduced fertilization and no inoculation for the leaf nitrogen content, each one promoting contents of 10.72 g of N/kg of plant and 10.68 g of N/kg of plant, respectively. However, plants from treatments with reduced fertilization and without inoculation and with MZT10M1 and MZT10M12 presented the lowest shoot dry matter production values.

Inoculation with MKC2M3, MLT8M19 and Ab-V5 promoted lower leaf nitrogen levels in the plants, which was even lower than the treatment with reduced fertilization and no inoculation.

The highest values of foliar phosphorus content were obtained with complete mineral fertilization, which induced a P content of 3.62 g/kg of plant (Figure 8). This treatment differed statistically from treatments with inoculation and control, showing an increase of 61.61% compared to the latter. The inoculation of seedlings with MET12M2 allowed an increase of 43.75% of the phosphorus content in leaves compared to the plants submitted to reduced fertilization and no inoculation. The plants from the inoculated treatment presented contents of 3.22 g of P/kg, and those with reduced fertilization and no inoculation showed 2.24 g of P/kg. In addition, the inoculation of MET12M2 allowed the highest P content when compared to the other tested isolates. However, all isolates improved the P uptake in plants compared to the control without inoculation and with reduced fertilization, and no statistical differences were observed between the P content in plants treated with isolates MKC2M3, MLT8M19, MZT10M1, MZT10M12, and the Ab-V5 strain.

More effective results were obtained for the accumulated nitrogen (Figure 8E) and phosphorus (Figure 8F), for which the highest values were obtained through the application of complete mineral fertilization, with an accumulation of 144.76 mg of N/plant and 43.55 mg P/plant, respectively. These values correspond to an increase of 352.66% in nitrogen accumulation and 550% in phosphorus accumulation compared to the control. The application of complete fertilization was significantly different from all other treatments. The most significant accumulation of N and P was observed after inoculation of MET12M2, with values of 121.82 mg N/plant and 36.57 mg P/plant, corresponding to an increase of 280.93% and 445, 82% in the accumulation of N and P, respectively, compared to the control. Other isolates showed intermediate N and P accumulation levels and significantly influenced this variable compared to the control.

**Figure 9:**
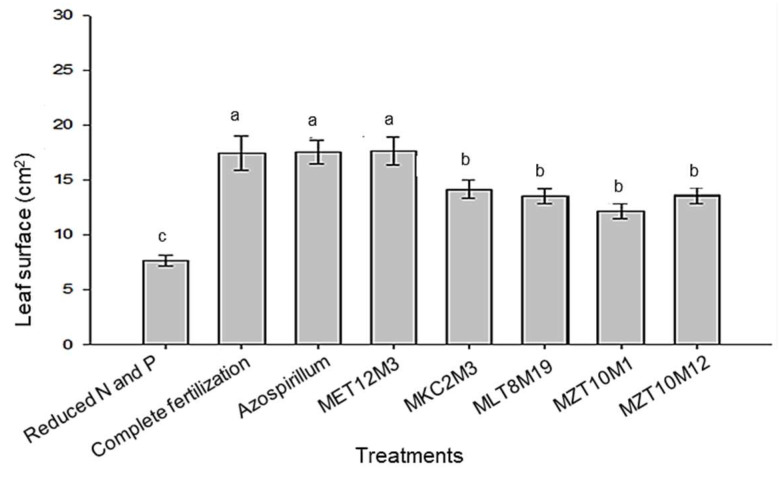
Leaf area of plants inoculated or not with in vitro selected endophytic bacteria. Means represented by the same letter do not differ significantly by the Scott–Knot test at 5% probability.

The leaf area (Figure 9) was also significantly (*p* < 0.05) influenced by the treatments. Higher values for this variable were obtained in treatments with complete fertilization and with inoculation of Ab-V5 or MET12M2. Other isolates showed intermediate values for this variable, all significantly different from the control.

**Figure 10:**
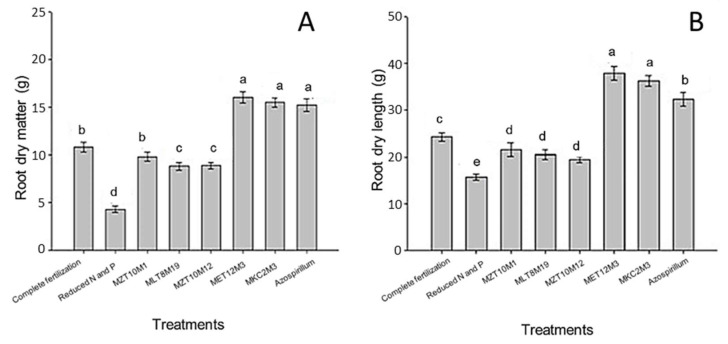
Root dry matter (**A**) and root length (**B**) in uninoculated plants and plants inoculated with in vitro selected endophytic bacteria. Means represented by the same letter do not differ significantly by the Scott–Knot test at 5% probability.

Root dry matter (Figure 10A) and root length (Figure 10B) were also significantly influenced by the treatments with fertilization and inoculation (*p* < 0.05). The highest root production (in weight and height) was observed with the inoculation of MKC2M3 and strain Ab-V5, which showed on average 16.06, 15.51, and 15.23 g of roots and 37.9, 36.3, and 32.43 cm of roots, respectively. These values correspond to an increase of 274.36%, 261.54%, and 255.01% in root weight and 141.25%, 131.06%, and 106.43% in root length with the inoculation of MET12M2, MKC2M3 and Ab-V5, respectively.

## 4. Discussion

Results obtained in auxin production tests showed the ability of 130 bacterial isolates to synthesize this phytohormone, with the best results observed for isolates 44, MZT10M1, MQT16M1, 30, MQT4M1, 23 and MET12M2.

In general, the amount of produced auxin can vary among the isolates and depends on the culture conditions. Very high productions of this plant regulator by different bacterial isolates have already been described. Assessing the growth promotion of lettuce by *Rhizobium leguminosarum* strains, 171.2 µg/mL of IAA production was reported [70]. However, auxin production at very high levels may inhibit plant growth [70,71].

Testing isolates belonging to genera *Pantoea*, *Pseudomonas*, *Microbacterium*, *Paenibacillus,* and *Chryseobacterium*, Verma et al. [72] observed levels of auxin production that varied from 6.6 (*Chryseobacterium* sp.) to 47.06 µg/mL (*Pantoea hericii*). The authors reported that inoculation with strains that were greater and low producers of auxin induced an increase in root length and the formation of root hairs. The same was observed in assays with different isolates of *Bacillus* spp. that showed an average production of 1.36 to 19.42 µg/mL of auxin [39].

Specifically, in strawberry plants, bacteria from the genera *Bradyrhizobium*, *Azospirillum*, *Enterobacter*, and *Burkholderia* produced from 0.3 to 5 µg/mL and were able to promote growth [38,73].

However, in addition to the plant-growth-promoting abilities of each bacterial isolate [38,71] and cultivation conditions, the levels of auxin production are variable depending on the presence of the substrate and the growth phase of the microorganism, pH, presence of organic acids, metals [74,75,76] and presence of the precursor tryptophan [77].

During the interaction with plants, root exudates allow the supplementation of tryptophan in the rhizosphere, which microorganisms can use to synthesize auxin [78,79]. Likewise, rhizosphere microorganisms can stimulate tryptophan exudation by plants and increase the expression of genes related to its transport and synthesis [80].

The bacterial isolates obtained in this work showed an ability to solubilize phosphate in vitro at different levels, with the highest levels of solubilization observed with the isolates MLT8M19, MKC2M3, MET12M2, 133, MET16M10 and MIT13M12.

Bacteria from different genera have been described as phosphate solubilizers, including members of *Acinetobacter*, *Paenibacillus*, *Rhizobium* [81] *Pseudomonas*, and *Enterobacter* [82]. However, the capacity and efficiency of phosphate solubilization appear to be strain-dependent [82,83], reinforcing the need to search for isolates capable of solubilizing this element in the soil. This ability has been linked to the production of different organic acids [81,84,85,86], with gLuconic acid being the most frequently observed [21,82].

The type of organic acid produced by each species has been reported as an essential factor for the efficiency of phosphate solubilization, and the simultaneous production of different acids can increase the capacity of a given isolate to solubilize this nutrient [81,83]. The solubilization of inorganic phosphate and the mineralization of organic phosphate seem to be common features among plant growth-promoting bacteria [87,88]. The biosynthesis of organic acids has been mainly related to the solubilization of inorganic P, while phosphatase activity plays an important role in the mineralization of organic P [89]. Whether the bacterial isolates selected are able to solubilize organic phosphate in different soil conditions remains to be elucidated in future field studies.

Additionally, bacteria can also solubilize phosphates by releasing H^+^ protons resulting from NH_4_ assimilation and H^+^-ATPase activity during ATP hydrolysis [90,91] or through the biosynthesis of exopolysaccharides [84,86] and siderophores with high affinity for iron [53,92]. However, these traits were not evaluated in this study.

In general, organic and inorganic phosphorous is available for plants only in low amounts due to its chemical affinity with Ca^2+^, Fe^2+^ and Al^2+^ ions [93,94]. Therefore, phosphorus is usually found in its insoluble forms, not available to plants, as it is very stable, of low reversibility and has a solubility that only decreases over time [94]. Thus, the use of phosphorus-solubilizing microorganisms may potentially become an important strategy in sustainable agriculture.

Only 59 bacterial were able to asymbiotically fix nitrogen. This test is qualitative and does not allow inferences in terms of the nitrogen amounts fixed by each isolate.

The use of nitrogen-fixing microorganisms in agriculture is a relevant alternative to nitrogen fertilizers, as the atmospheric assimilation is limited, requiring transformation to a combined form [95,96]. Thus, large amounts of nitrogen fertilizers are needed in food production, increasing production [97] and environmental costs [95,98].

The inoculation of strawberry seedlings with bacterial isolates previously selected in vitro caused changes in plant growth. The shoot dry matter and the number of leaves were significantly influenced by the treatments, especially by the inoculation of MET12M2. This result is particularly interesting, considering that only 30% of the total nitrogen and phosphorus composition of the nutrient solution was added to the inoculated treatments. Other bacterial isolates provided minor or intermediate increases in shoot dry matter, but all treatments differed statistically from the control

Concerning the leaf nitrogen content, the inoculation with MET12M2 was not significantly different from the control for leaf nitrogen content. However, the plants in the control group and those with MZT10M1 and MZT10M12 showed the lowest shoot dry matter production values. It can be inferred that this is a compensation mechanism for the low availability of nitrogen by decreasing dry matter production and increasing or maintaining nitrogen. Lower leaf nitrogen contents may be related to nutrient dilution in plant tissues with higher dry matter production.

Inoculation with MKC2M3, MLT8M19 and the control Ab-V5 promoted lower leaf nitrogen levels at levels lower than the control. Competition for nutrients between rhizospheric microorganisms and plants has been widely reported. Above all, microorganisms can compete with plants when facing low levels of certain nutrients [95]. The results of nitrogen contents observed in this study for the isolates mentioned above can potentially depend on this phenomenon. It is estimated that the decrease in nitrogen content in plants may also be related to the dilution of the nutrient in tissues during plant growth.

Other studies have shown that inoculation with growth-promoting bacteria can control the plants’ efficiency in the use of nitrate present in soil. Pii et al. [99] observed that the microorganism *A. brasilense* is able to reduce the use of nitrogen in the form of NO_3_ by mayse, in addition to changing the dynamics of plant genes involved in its use. The inoculation of some isolates may have affected the nitrate in the solution, explaining lower levels than those observed for the control.

The inoculation of seedlings with MET12M2 allowed the most significant increase in the foliar phosphorus content compared to the control. However, all isolates promoted, at different levels, such an increase in phosphorus uptake.

More significant results were obtained for the accumulated nitrogen and phosphorus, with the highest accumulation observed after inoculation with MET12M2, which induced an increase of 280.93% and 445.82% in nitrogen and phosphorus, respectively, compared to the control.

Inoculation with Ab-V5 and MET12M2 also increased the leaf surface. Similar results were observed by Erdogan et al. [100], who reported that the inoculation of *Bacillus* and *Pseudomonas* promoted a significant increase in leaf surface in grapevines, and by Rodrigues et al. [101] after inoculation of sugarcane with isolates of *Enterobacter*, *Pantoea,* and *Klebsiella*. In strawberries, an increase in the number and surface of leaflets and roots was observed after inoculation with *Bacillus methylotrophicus* isolate M4-96 [42]. Increasing the leaf surface is especially interesting because it has a direct link with the light harvested by the plants and, consequently, photosynthesis.

Dry matter and root length were also significantly influenced by the fertilization and inoculation treatments. The highest root production (in weight and length) was observed in treatments with inoculation of MET12M2, MKC2M3 and Ab-V5.

Data showed that inoculation with auxin-producing microorganisms increased the roots’ length and the induction of lateral roots.

Auxins promote plant growth and stimulate the H^+^-ATPase electrogenic pump activity in the plasma membranes at low concentrations, stimulating root growth [102]. In this study, even though the auxin production varied among isolates, the amounts produced were able to increase the roots in the inoculated plants. With the inoculation of *Enterobacter* and *Bacillus* isolates capable of producing from 1.89 to 19.37 µg/mL, Pereira et al. [39] observed a maximum increase of 184.88% in the dry weight of roots. Surprisingly, the highest increases in root length and root dry weight obtained in this work were not obtained after inoculation with the isolate that showed the most significant in vitro auxin production. This result may be related to the interaction of isolates with the plant species, especially through bacterial survival and multiplication and the stimulation of plant defense responses [103]. Moreover, auxin in excessive levels can promote the opposite effect by inhibiting root growth [70].

Auxin production is not the only mechanism deployed by growth-promoting bacteria. Working with *Bacillus* spp. in the model plant *Arabidopsis thaliana*, Hossain et al. [104] observed that these bacteria were able to produce volatile organic compounds independent of the auxin, ethylene, and jasmonate production systems. They were able to induce root development, showing the occurrence of other types of signaling by which bacteria can stimulate root growth. A further report showed the capacity of growth-promoting bacteria to induce the production of auxins by plants [105].

A beneficial effect of inoculating strawberry seedlings with MET12M2 was shown. Interestingly, this isolate did not show the highest phosphate solubilization or auxin production in vitro. However, its results were among the five best for all in vitro tests.

The effect of PGPB on plant dry matter accumulation was already *Bacillus*, *Pseudomonas*, *Enterobacter*, *Burkholderia*, gL*uconacetobacter*, and *Azospirillum* [38,45,106,107]. Several reports highlighted the benefits of PGPB. After inoculating *Pseudomonas* and *Bacillus* isolates in strawberries, Esitken et al. [107] observed an increase in the dry matter weight, yield, nitrogen and phosphorus contents in the leaves, with an increase of 471.43% in the level of foliar phosphorus. In the same plant, after the inoculation of *Azospirillum*, *Enterobacter*, and *Burkholderia* spp. And mineral fertilization containing 50% of the nitrogen requirement, Andrade et al. [38] observed an increase of up to 96% in plant dry matter when compared to the control. In that same work, variables such as root length and dry matter were also positively influenced by inoculation.

The sequencing of the 16S rDNA gene allowed the identification of taxa known to include PGPB. Bacteria belonging to the genera *Bacillus* [38,39,42,100,104], *Enterobacter* [73,82,101] and *Paenibacillus* [72,81] have been described as PGPB, including in strawberry [44], with the ability to fix nitrogen asymbiotically, as well as to produce auxins and solubilize phosphate. Surprisingly, MET12M2, which showed the greatest potential for promoting growth in strawberry plants, belongs to the genus *Brevibacillus*. Little is known about members of this genus as PGPB. The inoculation of Brevibacillus induced the growth of cotton through the ability to fix nitrogen and produce auxins [108]. To our knowledge, this is the first report that bacteria belonging to the genus *Brevibacillus* sp. solubilize phosphate and promote growth in strawberry plants.

This work represents an additional step in the research of new biofertilizers for strawberry production. *Brevibacillus fluminis* MET12M2 shows potential for direct application in the field or during the production of strawberry plantlets, facilitating integrated strawberry production. More studies are needed, however, to test the effect of inoculation with this strain on strawberry plants under different environmental conditions.

## 5. Conclusions

Endophytic bacteria isolated from strawberry leaves and roots showed different capacities of biosynthesizing auxins, fixing nitrogen and solubilizing phosphate in vitro. The most promising isolates belonged to the genera *Bacillus*, *Enterobacter* and *Paenibacillus* and to the species *Brevibacillus fluminis*. Isolates selected in vitro showed different effects on strawberry growth. *Brevibacillus fluminis* MET12M2 promoted more significant effects on strawberry growth, increasing variables such as shoot dry matter, number of leaves, leaf surface, root length and dry matter, as well as total nitrogen and phosphorus accumulated in the dry matter.

## Figures and Tables

**Table 1:** Identification based on 16S rDNA partial sequences of strains isolated from strawberry leaves and roots that were efficient in in vitro tests.

Strain	Origin	Identification	GenBank Accession Number
MET12M2	Roots	*Brevibacillus fluminis*	OM455371
MKC2M3	Leaves	*Bacillus* sp. Cereus clade	OM455372
MLT8M19	Roots	*Bacillus* sp. Cereus clade	OM455373
MZT10M1	Roots	*Enterobacter* sp.	OM455374
MZT10M12	Roots	*Paenibacillus* sp.	OM45537

**Table 2:** Asymbiotic nitrogen fixation by strawberry endophytic bacteria (qualitative test).

Strain	BNF	Strain	BNF	Strain	BNF	Strain	FBN	Strain	BNF
60	−	139	−	135	−	80	−	43	−
MQT10M16	+	56	+	MZT3M14	−	MHT7M	−	88	−
MLT14M7	−	MKC2M3	++	115	+	184	−	101	−
MLT14M7	−	MHT8M6	+	70	++	16	−	51	−
63	−	MNT10M4	−	117	++	111	−	100	−
MFT4M8	−	MZT10M1	+	77	++	137	−	138	−
28	−	53	+	87	−	123	−	MZT3M5	−
40	−	125	+	MQT6M1	−	MVT12M8	−	132	−
MZT10M12	+	68	−	122	++	MXT7M12	++	27	++
108	−	128	−	33	+	30	−	29	−
8	+	17	+	MLT8M19	++	116	+	152	−
24	+	MET12M2	++	57	+	94	++	44	−
9	+	MGT5M13	++	MTT9M2	−	179	−	26	++
13	+	92	++	104	−	MTT12M4	−	133	−
MST12M1	+	39	++	75	−	MIT13M12	−	MXT12M2	++
1	−	6	−	MTT16M8	++	86	+	127	+
50	−	34	++	14	−	MRT15M8	−	182	−
22	−	119	+	129	−	MTT12M4	−	181	−
MST6M3	+	84	++	21	−	MQT4M1	−		
MSC2M5	+	112	+	MQT16M1	−	124	−		
MDT12M6	−	93	+	MET16M10	++	143	−		
MDT7M5	+	12	−	MIT3M11	++	73	++		
47	−	2	+	79	−	136	−		
MST15M4	+	MTT5M10	++	109	++	MXT16M12	−		
MDT10M17	+	MIT15M14	++	MGT3M15	+	MGT3M6	−		
MIT11M11	+	107	−	69	−	54	−		
MFT5M11	+	69	−	107	−	MST13M1	−		
31	+	131	−	121	++	15	−		
23	−	151	++	MAT16M4	−	57	−		
154	−	58	+	MPT6M12	−	113	−		

Symbols observed refer to the classification of isolates according to the thickness of the aerotaxic film formed in NFb medium: − = no halo formation; + = thin halo (1mm); ++ = very thick halo (>1 mm).

## Data Availability

Data are available at http://purl.org/phylo/treebase/phylows/study/TB2:S29343.

## Supplementary Materials

The following supporting information can be downloaded at: https://www.mdpi.com/article/10.3390/microorganisms10122341/s1, Table S1: Selection of isolates obtained from strawberry roots and leaves based on their ability to produce auxin, solubilize phosphate and fix nitrogen asymbiotically. Principal component analysis.
