# Supplementary material for: The Friend Within: Endophytic Bacteria as a Tool for Sustainability in Strawberry Crops
Source: Microorganisms. 2022 Nov 26;10(12):2341. doi: 10.3390/microorganisms10122341 (PMC9780916; doi:10.3390/microorganisms10122341)
Supplement: Supplementary file 1 [file microorganisms-10-02341-s001.zip › microorganisms-1958597-supplementary.pdf]

SUPPLEMENTARY DATA

Table S1: Selection of isolates obtained from strawberry roots and leaves based on their ability to produce auxin, solubilize phosphate and fix nitrogen asymbiotically. Principal Component Analysis.

Isolate	Score	BNF*	Group
MLT8M19	3,74	++	16
MET12M2	3,00	++	11
MZT10M1	2,78	+	18
127	2,57	+	3
MZT10M12	2,31	+	19
30	2,26	-	7
MKC2M3	2,08	++	15
MIT13M12	2,03	-	15
23	1,77	-	6
133	1,56	-	4
123	1,55	-	1
MGT3M15	1,55	+	10
MQT4M1	1,53	-	6
93	1,48	+	1
121	1,42	++	1
MSC2M5	1,32	+	1
MHT8M6	1,32	+	4
MQT16M1	1,26	-	17
MIT11M11	1,23	+	14
128	1,22	-	1
MFT4M8	1,20	-	1
56	1,08	+	10
MIT15M14	1,07	++	1
MQT10M16	0,94	+	1
MGT5M13	0,94	++	1
75	0,93	-	1
34	0,82	++	1
51	0,81	-	10
9	0,80	+	1
79	0,79	-	1
MNT10M4	0,78	-	1
MST15M4	0,77	+	1
MFT5M11	0,77	+	1
44	0,72	-	9
43	0,71	-	1
13	0,69	+	1
MGT3M6	0,67	-	13

Scores and group calculated based on the ability to produce auxin and solubilize phosphate of each isolate. *BNF: Biological nitrogen fixation. - : unable to fix nitrogen asymbiotically; + : able to fix nitrogen asymbiotically; ++ : formation of an aerotaxic halo greater than 1mm.

Table S1: – Selection of isolates obtained from strawberry roots and leaves based on their ability to produce auxin, solubilize phosphate and fix nitrogen asymbiotically. Principal Component Analysis.

Isolate	Score	BNF*	Group
50	0,66	-	1
139	0,64	-	1
MET16M10	0,58	++	12
69	0,56	-	1
136	0,49	-	1
17	0,48	+	1
80	0,46	-	1
153	0,44	-	1
MTT9M2	0,43	-	1
122	0,43	++	1
MDT10M17	0,42	+	1
12	0,42	-	1
28	0,39	-	1
84	0,36	++	1
29	0,32	-	1
137	0,32	-	1
113	0,26	-	1
73	0,25	++	1
119	0,23	+	1
1	0,17	-	1
117	0,16	++	1
MST13M1	0,14	-	1
22	0,13	-	1
63	0,11	-	1
92	0,05	++	1
MPT6M12	0,04	-	1
108	0,01	-	1
101	-0,01	-	1
94	-0,01	++	1
8	-0,02	+	1
184	-0,02	-	1
2	-0,04	+	1
MIT3M11	-0,04	++	1
14	-0,05	-	1
47	-0,07	-	1
MDT7M5	-0,07	+	1
MST12M1	-0,09	+	1
16	-0,10	-	1
6	-0,10	-	1
107	-0,15	-	1
104	-0,15	-	1
			1
87	-0,16	-	1

Scores and group calculated based on the ability to produce auxin and solubilize phosphate of each isolate. *BNF: Biological nitrogen fixation. - : unable to fix nitrogen asymbiotically; + : able to fix nitrogen asymbiotically; ++ : formation of an aerotaxic halo greater than 1mm.

Table S1: Selection of isolates obtained from strawberry roots and leaves based on their ability to produce auxin, solubilize phosphate and fix nitrogen asymbiotically. Principal Component Analysis.

Isolate	Score	BNF*	Group
21	-0,21	-	1
77	-0,23	++	1
100	-0,25	-	1
15	-0,27	-	1
88	-0,28	-	1
70	-0,30	++	1
115	-0,32	+	1
MST6M3	-0,34	+	1
65	-0,40	-	1
MDT12M6	-0,42	-	1
24	-0,43	+	1
116	-0,45	+	1
MXT16M12	-0,46	-	1
135	-0,54	-	1
86	-0,57	+	1
138	-0,60	-	1
MVT12M8	-0,62	-	1
MRT15M8	-0,72	-	1
154	-0,78	-	1
132	-0,79	-	1
MHT7M	-0,81	-	1
57	-0,84	+	1
MXT7M12	-0,85	++	1
27	-0,90	++	1
MTT5M10	-0,94	++	1
39	-1,01	++	1
MZT3M14	-1,02	-	1
179	-1,04	-	1
MXT12M2	-1,07	++	1
MTT12M4	-1,08	-	1
MAT16M4	-1,08	-	1
131	-1,10	-	1
68	-1,16	-	2
40	-1,19	-	8
125	-1,19	+	1
151	-1,21	++	1
54	-1,23	-	1
182	-1,33	-	2
124	-1,35	-	2
MZT3M5	-1,36	-	20
58	-1,56	+	1
112	-1,57	+	1

Scores and group calculated based on the ability to produce auxin and solubilize phosphate of each isolate. *BNF: Biological nitrogen fixation. - : unable to fix nitrogen asymbiotically; + : able to fix nitrogen asymbiotically; ++ : formation of an aerotaxic halo greater than 1mm.

Table S1: Selection of isolates obtained from strawberry roots and leaves based on their ability to produce auxin, solubilize phosphate and fix nitrogen asymbiotically. Principal Component Analysis.

Isolate	Score	BNF*	Group
33	-1,67	+	1
152	-1,67	-	1
111	-1,67	-	1
MTT16M8	-1,71	++	1
53	-1,78	+	1
143	-1,82	-	1
60	-1,85	-	1
26	-1,86	++	1
129	-1,87	-	1
109	-1,88	++	1
MLT14M7	-1,90	-	1
31	-1,99	+	1
181	-2,38	-	5
MQT6M1	-2,38	-	5

Scores and group calculated based on the ability to produce auxin and solubilize phosphate of each isolate. *BNF: Biological nitrogen fixation. - : unable to fix nitrogen asymbiotically; + : able to fix nitrogen asymbiotically; ++ : formation of an aerotaxic halo greater than 1mm.
